# Validation of extracellular miRNA quantification in blood samples using RT‐qPCR

**DOI:** 10.1096/fba.2019-00018

**Published:** 2019-07-01

**Authors:** Maria Fauth, Anett B. Hegewald, Lisa Schmitz, Dorothee J. Krone, Meike J. Saul

**Affiliations:** ^1^ Department of Biology Technische Universität Darmstadt Darmstadt Germany; ^2^ Prolytic GmbH Frankfurt/M Germany; ^3^ Institute of Pharmaceutical Chemistry Goethe Universität Frankfurt Frankfurt/M Germany

**Keywords:** biomarker, GLP, miRNA, plasma, RT‐qPCR, validation

## Abstract

Extracellular microRNAs (miRs) have been proposed as important blood‐based biomarkers for several diseases. Contrary to proteins and other RNA classes, miRs are stable and easily detectable in body fluids. In this respect, miRs represent a perfect candidate for minimal invasive biomarkers which can hopefully become a complement for invasive histological examinations of tumor tissue. Despite the high number of miR biomarker studies, the specificity and reproducibility of these studies is missing. Therefore, the standardization of pre‐analytical and analytical methods is urgently needed. Here, we validated miR analysis for RNA isolation and miR quantification by quantitative polymerase chain reaction (RT‐qPCR) based on good laboratory practice (GLP). Validation was carried out exemplarily on four miRs, which had already been described as potential biomarkers in previous studies. As basis for RNA analysis using RT‐qPCR, the Minimum Information for Publication of Quantitative Real‐Time PCR Experiments were applied and adapted on the analysis of circulating miRs from human plasma. In our study, we identified and solved several pitfalls from handling to normalization strategy in the analysis of extracellular miRs that lead to inconsistent and non‐repeatable data. Principles of GLP set a framework of experimental design, performance and monitoring to ensure high quality and reliable data. Within this study, we appointed first acceptance criteria for circulating miR quantification during validation which set standards for future miR quantification in blood samples.

**Abbreviations**
ath: *Arabidopsis thaliana*
cDNA: complementary DNA
cel: *Caenorhabditis elegans*
Cq: quantification cycle
CV: coefficient of variation
FFP: fresh frozen plasma
GLP: good laboratory practice
GTC: Guanidinium thiocyanate
hsa: *Homo sapiens*
LLOQ: lower limit of quantification
Med: median concentration
MIQE: minimum information for publication of quantitative real‐time PCR experiments
miRs: microRNAs
QC: quality control
R^2^: correlation coefficient
RT‐qPCR: quantitative reverse transcription polymerase chain reaction
SDS: Sodium lauryl sulfate

## 1 INTRODUCTION

MicroRNAs (miRs) are a family of small non‐coding RNAs (20‐22 nucleotides long) which have been emerged as major post‐transcriptional regulators of gene expression.^1^ Several thousand human miRs have been identified and it is believed that around two third of the human genome is directly regulated by miRs.^2^ Hence, a dysregulation of miRs is associated with several diseases, especially cancer.^3^ Although, most miRs were found intracellular, several miRs have been identified outside the cells in biofluids like blood or urine.^4^ These so‐called circulating miRs are incorporated in extracellular vesicles or associated with proteins like argonaut protein 2 (Ago2) which leads to a prolongation against rapid RNase degradation and thus to a high stability of the miRs.^5^, ^6^, ^7^ Beside their potential regulatory function, such circulating miRs are attributed to reflect disease physiology and/or treatment response and therefore, represent a promising non‐invasive diagnostic, prognostic, or predictive tool as biomarkers.^8^, ^9^

Although, miRs represent a very promising non‐invasive biomarker, there are some reservations. Over the last years, hundreds of novel miR biomarkers were identified for different diseases but the reported biomarkers are largely nonspecific and associated with a wide range of conditions and outcomes.^10^ Little agreement and overlap has been observed between nearly identical studies.^9^, ^10^ A critical point for this variability of miR biomarker studies is the lack of a robust, fast and valid diagnostic assay. This is partially due to variability of the analytical methods, sample collection, RNA extraction, and storage conditions.^9^, ^10^ This lack of standardization motivated us to develop a miR analytic method using quantitative reverse transcription polymerase chain reaction (RT‐qPCR) in unfractionated blood samples and plasma‐derived extracellular vesicles, which is validated for the first time in terms of good laboratory practice (GLP) guidelines, a quality system which examines the organization processes of pre‐clinical investigations.

With this principle, we appointed first acceptance criteria for circulating miR quantification during validation which set standards for future extracellular miR quantification.

## 2 MATERIALS AND METHODS

### 2.1 Plasma and serum samples

Plasma and serum samples were acquired commercially by Biotrend Chemikalien GmbH, Cologne, Germany. Fresh plasma and serum samples were processed from the freshly collected blood of a volunteer. After three times inverting the blood in the S‐Monovettes® it was kept on ice. Sarstedt S‐Monovette® plasma tubes were centrifuged at 2000× *g* and 4°C for 20 minutes. The blood collected in Sarstedt S‐Monovette® serum‐gel tubes was left on ice in vertical position for 30 minutes for coagulation before centrifugation at 2500× *g* and 4°C for 20 minutes. Samples were transferred as 10 µL aliquots into new sterile 1.5 mL tubes (Eppendorf AG, Hamburg, Germany) and stored at −80°C. Hemolytic plasma samples were not considered in our analysis due to the artificial increasing of the Cq (quantification cycle)‐values which would lead to pre‐analytical variations.^11^ All experiments with human blood samples of healthy donors were performed in compliance with the ethical standards according to the guidelines for good clinical practice.^12^ Informed consent was obtained from the participant.

### 2.2 Total RNA extraction

RNA was isolated from plasma or serum following the phenol/guanidinium thiocyanate (GTC)‐based extraction method of Chomczynski & Sacci (1987)^13^ with slight modifications of the protocol involving usage of 9.5% sodium lauryl sulfate (SDS) (AppliChem GmbH, Darmstadt, Germany) instead of sarcosyl for the lysis buffer. Ten microliter serum or plasma samples were thawed slowly on ice. A quantity of 420 µL lysis buffer which consists 200 µL extraction buffer (150 mmol/L sucrose (AppliChem GmbH, Darmstadt, Germany), 10 mmol/L sodium acetate (AppliChem GmbH, Darmstadt, Germany), pH 6.5), 20 µL SDS (20%) and 200 µL guanidinium thiocyanate (GTC, 6M) was added to lyse proteins and lipid complexes before adding 5 µL of synthetic ath (*Arabidopsis thaliana*)‐miR‐159a (5´‐UUUGGAUUGAAGGGAGCUCUA‐3'; 200 nmol/L, Sigma Aldrich, Darmstadt, Germany) or cel (*Caenorhabditis elegans*)‐miR‐39‐3p (5´‐UCACCGGGUGUAAAUCAGCUUG‐3'; 200 nmol/L, Sigma Aldrich, Darmstadt, Germany). Non‐human miRs were selected as internal standard to compensate technical and methodical variations.^14^ After extraction of RNA with 200 µL phenol (waterlogged) (AppliChem GmbH, Darmstadt, Germany) and 200 µL chloroform: isoamyl alcohol (1:24) (AppliChem GmbH, Darmstadt, Germany) the RNA was separated from DNA and proteins remaining in the organic phase by centrifugation for 5 min at 4°C and 13,300 x g in Heavy Phase Lock Gel Tubes^TM^ (Quantabio, Beverly, USA). The aqueous phase of about 500 µL containing total RNA was transferred into 2 mL LoBind reaction tubes (Eppendorf AG, Hamburg, Germany) and precipitated by 1/10 volume of sodium acetate (3 mol/L) (AppliChem GmbH, Darmstadt, Germany) and three times volume of ethanol (AppliChem GmbH, Darmstadt, Germany). The co‐precipitant GlycoBlue^TM^ (Invitrogen, Carlsbad, California, USA) was added to increase the precipitation efficiency. After precipitation, the pellet was washed and resuspended in 20 µL RNase‐free water. Samples were stored at −80°C until further processing. All concentrations of synthetic miRs impaled in samples reported in the results correspond to the concentration in the final resuspension volume of 20 µL of RNase‐free water at the end of RNA extraction. Additionally, we isolated total RNA by miRNeasy Mini Kit (Qiagen, Hilden, Germany) according to the manufacturer's instructions.

### 2.3 Quantification of miRs by RT‐qPCR

The miScript II RT Kit (Qiagen, Hilden, Germany) was used to generate miR first‐strand complementary DNA (cDNA) out of 2 µL of total RNA solution according to the manufacturer´s instructions. A fixed volume of total RNA instead of equal quantities of RNA was used due to the interference effects of phenol in optical investigations of RNA.^15^ The cDNA was diluted 1:2 and then used for qPCR of miRs with miScript SYBR® Green PCR Assay (Qiagen, Hilden, Germany) on a QuantStudio™ Flex Real‐Time PCR System (ThermoFisher Scientific, Waltham, USA) and a StepOne Real‐Time PCR System (Applied Biosystems, ThermoFisher Scientific, Waltham, USA) according to the manufacturer's instructions. The primer Hs_miR‐382_2 (MS00031836; Qiagen, Hilden, Germany) was used for specific amplification of hsa (*Homo sapiens*)‐miR‐382‐5p. The expression was normalized to ath‐miR‐159a using the primer At_miR‐159a_1 (MS00074871; Qiagen, Hilden, Germany). RT‐qPCR of hsa‐miR‐146a‐5p, hsa‐miR‐155‐5p and hsa‐miR‐451a was performed using Hs_miR‐146a_1 (MS00003535; Qiagen, Hilden, Germany), Hs_miR‐155_2 (MS00031486; Qiagen, Hilden, Germany) and Hs_miR‐451_1 (MS00004242; Qiagen, Hilden, Germany) primer. The expression of miR‐146a‐5p, miR‐155‐5p and miR‐451a was normalized to cel‐miR‐39‐3p using the specific primer Ce_miR‐39_1 (MS00019789; Qiagen, Hilden, Germany).

The concentrations of miRs in isolated total RNA from human plasma and serum were determined by a standard calibration curve over five logarithmic units (based on MIQE Guidelines, 2009), wherein the calibration standards were processed according to the phenol/GTC method. Cq (quantification cycle)‐values were calculated by automatic Cq algorithm and measured by RT‐qPCR. For calibration standards synthetic hsa‐miR‐146a‐5p (5'‐UGAGAACUGAAUUCCAUGGGUU‐3'; Sigma Aldrich, Darmstadt, Germany), hsa‐miR‐155‐5p (5'‐UUAAUGCUAAUCGUGAUAGGGGU‐3'; Sigma Aldrich, Darmstadt, Germany), hsa‐miR‐382‐5p (5´‐GAAGUUGUUCGUGGUGGAUUCG‐3'; Sigma Aldrich, Darmstadt, Germany) and hsa‐miR‐451a (5´‐AAACCGUUACCAUUACUGAGUU‐3'; Sigma Aldrich, Darmstadt, Germany) were prepared and diluted in RNase‐free water. All samples were evaluated in duplicates, and all runs included non‐template controls (NTC ≥ 37 Cq) for each miR. Melting curve analysis was used to assess the specificity of the amplified product. For normalization synthetic non‐human miRs cel‐miR‐39‐3p (5'‐UCACCGGGUGUAAAUCAGCUUG‐3'; 200 nmol/L; Sigma Aldrich, Darmstadt, Germany) or ath‐miR‐159a (5'‐UUUGGAUUGAAGGGAGCUCUA‐3'; 200 nmol/L; Sigma Aldrich, Darmstadt, Germany) were added to plasma samples and to each calibration standard. The calculation ∆Cq = Cq (analyte) ‐ Cq (internal standard) was applied to normalize Cq‐values. Concentration of miRs was determined using standard calibration curve (plotted logarithmic initial amount of dilution series of miRs against Cq‐values) by following calculation: $c\left[\frac{\mathrm{pmol}}{l}\right]=10^{\frac{\Delta Cq-intercept}{\mathrm{slope}}}$

### 2.4 Data analysis

All results are presented as mean with standard error of the mean (SEM) of at least two independent experiments. Differences are considered as significant by unpaired *t* test of GraphPad Prism 6 when *P* is < 0.05 (indicated as *P < 0.05, **P < 0.01, ***P < 0.001, ****P < 0.0001).

### 2.5 Method validation

Validation of an analytical method should be generally performed for each species, matrix and analyte concerned, ie each miR to be investigated. To evaluate the validity of the analytical method for miR quantification by RT‐qPCR, the validation was carried out referring to the guidelines for the analysis of genetically modified organisms (GMO) and for the quantification of DNA in food, according to ISO 5725:1994. Following parameters were considered for the validation:

#### 2.5.1 Linearity of standard calibration curve

Linearity of quantification method must be within the calibration range of measurement. The measuring range of the standard calibration curve include the smallest accurately measurable concentration standard (lower limit of quantification, LLOQ).^16^ Linearity of standard calibration curve was evaluated by preparing and measuring three standard calibration curves on three different days. The back‐calculated concentration values should be within ± 30% of the nominal value.^16^ The standard curve is plotted as ΔCq vs logarithm of template concentration (Figure S1). The conversion of ΔCq values into concentrations based on the intercept and slope of standard curve and is calculated as $C\left[\frac{\mathrm{pmol}}{l}\right]=10^{\frac{\Delta Cq-intercept}{\mathrm{slope}}}$. The amplification efficiency of the method is determined by the slope of the standard curve as $E={(10}^{\left(\frac{-1}{\mathrm{slope}}\right)}-1)*100$. Ideally, the slope is between −3.6 and −3.1, which corresponds to an efficiency of 90%‐110%.^17^, ^18^ For samples where extraction of nucleic acids may be difficult, a slope up to −4.1 is acceptable.^17^

#### 2.5.2 Sensitivity

Sensitivity of RT‐qPCR was assessed by determining the LLOQ, which defines the smallest accurately measurable concentration of miRs.

#### 2.5.3 Recovery

The isolation method has to extract the amount of nucleic acids from sample material required for subsequent RT‐qPCR analysis.^17^ The recovery was determined by comparing the responses of processed samples, which were spiked with a defined miR concentration either before or after RNA isolation, measured by subsequent RT‐qPCR analysis.

#### 2.5.4 Precision

The precision, as a parameter to describe the repeatability and reproducibility of an analytical method, was analyzed for the quantification of nucleic acids. Repeatability or intraday‐precision refers to the precision of one sample repeatedly prepared and analyzed in one run.^19^ Reproducibility or interday‐precision defines the variation in test results of different runs or between different laboratories.^19^ In this experimental setup the repeatability was evaluated by preparing and analysing one plasma in triplicates. Reproducibility was assessed by determination of the variance of three independent runs (n = 3 per day, ie, n = 9 for 3 days). The test conditions must be kept the same. Variations of 25% for intraday‐precision and 35% for interday‐precision are acceptable for the entire dynamic measuring range, except the limit of quantification, where the variation can be higher.^16^, ^17^ The precision of the method was evaluated by the coefficients of variation (CV) of the measured values for the quality control samples (QCs). The concentration of QCs varies depending on circulating miR concentration in plasma, ie the higher the circulating miR‐concentration, the higher the concentration of the QC is adjusted.

$$\text{CV}\left(\%\right)=\frac{\text{Standard deviation of the calculated concentration}}{\text{Mean value of the calculated concentration}}*100$$

#### 2.5.5 Accuracy

Uncertainty of measurement, ie the deviation of the measured concentration from the nominal concentration of ±25% is acceptable for the detection of nucleic acids.^16^, ^17^, ^20^ Intraday‐accuracy refers to deviation between the measured concentrations of one sample repeatedly prepared and analyzed in one analytical run to the nominal concentration. Interday‐accuracy defines the mean of deviations between the measured concentrations of one sample repeatedly analyzed in different analytical runs or between different laboratories. The uncertainty of measurement is shown as accuracy in percent. The evaluation of the accuracy of calculated concentrations is also determined by quality controls.

$$\text{A}\left(\%\right)=\frac{\text{Calculated concentration}-\text{target concentration}}{\text{Target concentration}}$$

#### 2.5.6 Matrix effect

For evaluation of matrix effect water and plasma were spiked with miR at three different concentrations. Cq‐values of miR in the water QC was compared with the Cq‐values in matrix matched QC at the same concentration level.

#### 2.5.7 Stability

Long‐term storage stability of hsa‐miR‐155‐5p and hsa‐miR‐451a in plasma, as RNA isolate in RNase‐free water and as cDNA derivate were investigated. Plasma and RNA isolate were stored at approximately −80°C, while the cDNA derivate was stored at approximately −20°C. The stability analysis of RNA isolate and cDNA derivate was performed after one day of RNA isolation, after 7 days, after one month and after 4 months of storage. Stability of RNA in plasma was investigated after one month and 4 months of storage at approximately −80°C. Plasma samples were processed freshly before storage to determine the reference concentration of non‐stored miR plasma samples. Stability investigations were performed using freshly prepared standard calibration curves and quality control samples.

## 3 RESULTS

### 3.1 Influence of the RNA extraction process on the miR recovery rate

**Figure 1:**
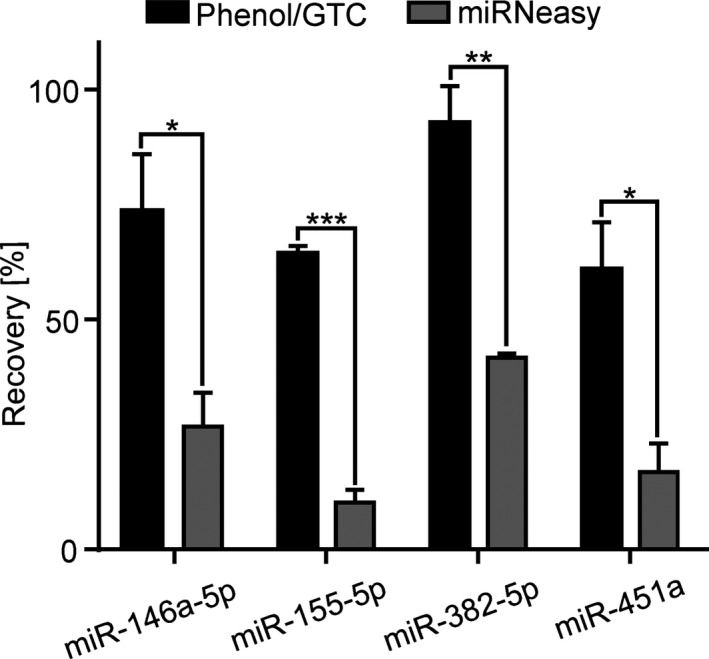
Comparison of miR recovery using different RNA isolation methods. Concentrations of miRs were quantified from 10 µL K3‐EDTA plasma spiked with synthetic miR‐146a‐5p, miR‐155‐5p, miR‐382‐5p, or miR‐451a (2.5 nmol/L) either before or after RNA extraction. As internal standard cel‐miR‐39‐3p and ath‐miR‐159a (50 nmol/L) were used for normalization. Internal standard was spiked in all samples before RNA isolation. Recovery of miRs isolated by Phenol/GTC RNA extraction method and by commercial miRNeasy RNA isolation kit were calculated by ratio between the measured concentration (spiked before RNA isolation) and concentration (spiked after RNA isolation) and is given as mean + SEM of three independent RNA isolations on two independent experimental days; *t* test, *P < 0.05, **P < 0.01, ***P < 0.001

We evaluated the recovery rate of miR extraction from human potassium‐3‐ ethylenediaminetetraacetic acid (K3‐EDTA) plasma samples using two different RNA extraction methods. We have chosen a phenol/GTC‐based RNA extraction method based on Chomczynski & Sacci (1987)^13^ coupled with acetate/ethanol RNA precipitation and a commercial available phenol‐based RNA extraction kit. We quantified miR‐146a‐5p, miR‐155‐5p, miR‐382‐5p, and miR‐451a which have been described as potential blood based biomarkers for different diseases^21^, ^22^, ^23^, ^24^ using miScript II RT Kit (Qiagen, Hilden, Germany). Recovery was determined by comparing the quantified concentration of human K3‐EDTA plasma samples spiked with 2.5 nmol/L synthetic miR‐146a‐5p, miR‐155‐5p, miR‐382‐5p, and miR‐451a either before or after RNA isolation. The ratio of both miR‐concentrations was used to calculate the recovery rate. For normalization we added 50 nmol/L synthetic non‐human miRs ath‐miR‐159a or cel‐miR‐39‐3p. We could show that the recovery of all analyzed miRs isolated by phenol/GTC based RNA extraction method is 44%‐54% higher than with column based RNA isolation, resulting in an overall miR recovery of 60%‐92% (Figure 1). Since miRs are often very low concentrated in the blood, a high recovery rate was particularly important to us. Therefore, we have opted for a phenol/GTC‐based RNA extraction method for our further miR quantification validation.

### 3.2 Pre‐analytical variances influence miR analysis

#### 3.2.1 Sample matrix

**Figure 2:**
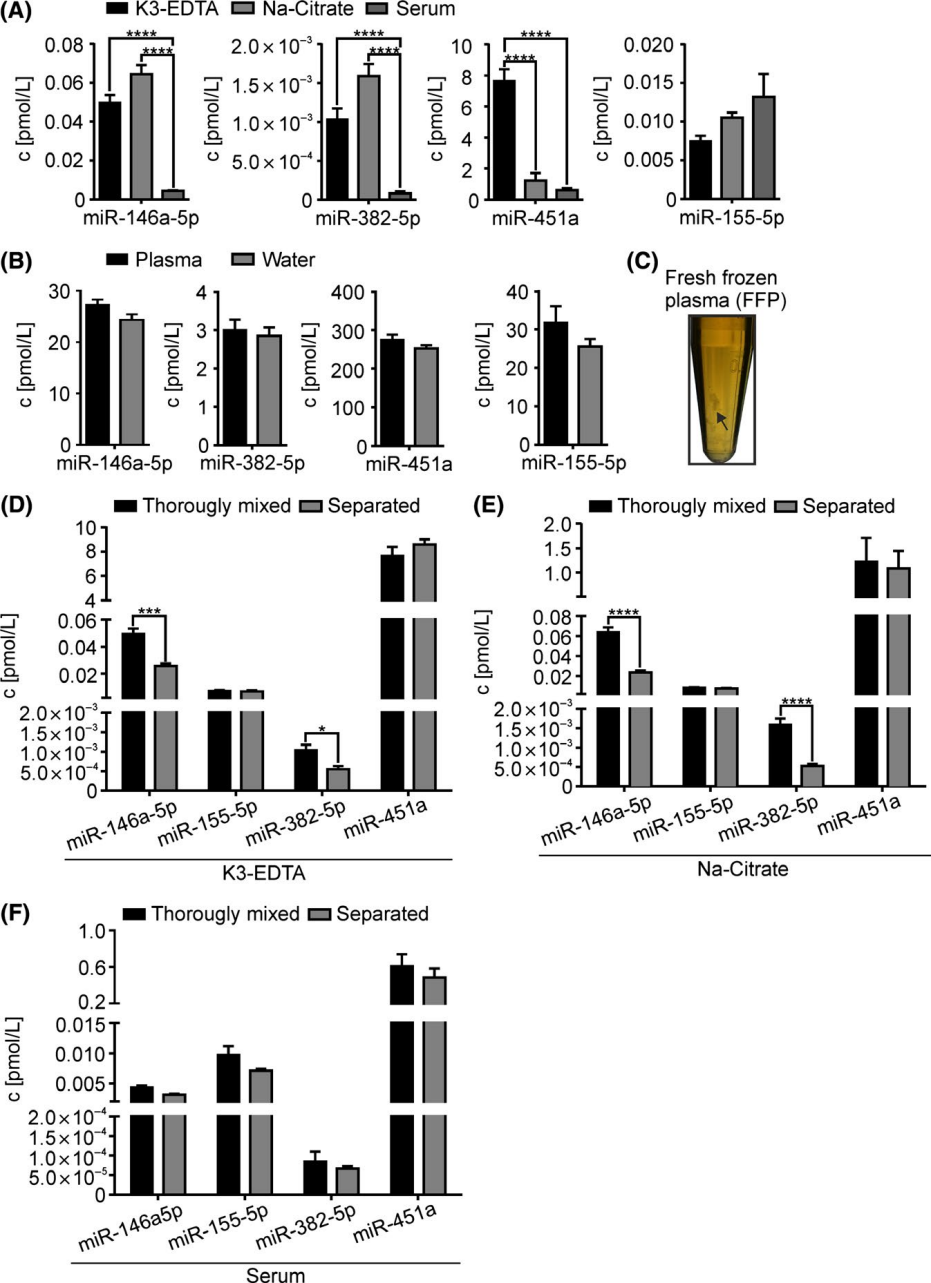
Effect of pre‐analytical variabilities on miR quantification results. A, Comparison of circulating miR‐146a‐5p, miR‐155‐5p, miR‐382‐5p, and miR‐451a concentrations isolated from 10 µL serum or plasma from one participant with additional consideration of the influence on miR quantification of the different anticoagulants K3‐EDTA and Na‐Citrate. B, The matrix effect of K3‐EDTA plasma on the quantification of miR‐146a‐5p, miR‐155‐5p, miR‐382‐5p, and miR‐451a by RT‐qPCR is illustrated by comparing the determined concentrations of miR in plasma and in water. Therefore, plasma and water were spiked with synthetic miR‐146a‐5p (25 pmol/L), miR‐155‐5p (2.5 pmol/L), miR‐382‐5p (2.5 pmol/L), and miR‐451a (250 pmol/L) and were processed by phenol/GTC RNA extraction method. C, Plasma stored at −80° and thawed slowly at 4°C. In this so called fresh frozen plasma (FFP) precipitates can be partially identified (black arrow). The extracellular concentrations of miR‐146a‐5p, miR‐155‐5p, miR‐382‐5p, and miR‐451a were quantified by RT‐qPCR in FFP with (D) K3‐EDTA or (E) Na‐Citrate as anticoagulant or (F) in serum processed like FFP. Samples were thoroughly mixed by vortexing and shaking or centrifuged to separate the precipitate. The extracellular miR‐concentrations of 10 µL of differently processed plasma or serum samples were compared. Concentrations of miRs are normalized using ath‐miR‐159a (50 nmol/L) or cel‐miR‐39‐3p (50 nmol/L) as internal standard and given as mean + SEM of (A) six, of (B) three independent RNA isolations or (E‐F) of two independent experimental days; *t* test, *P < 0.05, ***P < 0.001, ****P < 0.0001

When studying biomarkers in serum or plasma, different approaches of blood collection are often used, for example, the use of different anticoagulants. The matrix of differently prepared plasma can be variable and lead to deviations in miR quantification, termed as matrix effect.^16^, ^25^ It has been described that anticoagulants like Lithium (Li)‐Heparin can influence the quantification of miRs using RT‐qPCR.^26^ Therefore, we assessed the matrix effect of anticoagulants Li‐Heparin, K3‐EDTA and sodium citrate (Na‐Citrate) in plasma from one participant and as well the matrix effect of serum by quantifying extracellular miR‐146a‐5p, miR‐155‐5p, miR‐382‐5p, and miR‐451a. The matrix seems to have a negligible effect on measurement signal in quantification of miRs, excluding plasma with the anticoagulant Li‐Heparin (Figure S2). Residues of Li‐Heparin inhibit the enzymatic reaction of reverse transcription or qPCR.^27^ Since Cq‐values are directly proportional to the logarithm of ten to the concentration, the influence of the matrix must be characterized by the absolute miR quantity. The concentrations of miR‐146a‐5p, miR‐382‐5p, and miR‐451a are in any type of plasma significantly higher than in serum samples. No variations of miR‐146a‐5p and miR‐382‐5p were observed using K3‐EDTA or Na‐Citrate plasma. In contrast, there is a high difference in the level of miR‐451a in K3‐EDTA and Na‐Citrate plasma. Since hsa‐miR‐451a is considered as red blood cell related miR,^28^ the high miR‐451a level in K3‐EDTA plasma could be released by increased red blood cell vesiculation due to EDTA.^29^ We observed no significant matrix effect in serum and plasma samples on miR‐155‐5p quantification which indicates a dependence of matrix effect on the sequence of miRs being analyzed (Figure 2A). Overall, we have chosen to use K3‐EDTA plasma for miR quantification validation. Our results revealed that this is the most universally applicable matrix which stands in line with Tuck et al.^30^

Not only anticoagulants in the plasma matrix can influence the miR quantification, but also residuals from the RNA extraction process. To investigate this plasma related matrix effect, synthetic miR‐146a‐5p (25 pmol/L), miR‐155‐5p (2.5 pmol/L), miR‐382‐5p (2.5 pmol/L) and miR‐451a (250 pmol/L) were either spiked in water or in plasma samples. We selected lower spike‐in concentrations depending on endogenous miR concentration in plasma to cover the low concentration range of circulating miRs. Our results showed no significant difference in miR quantification between analysis using water and plasma matrix (Figure 2B). Therefore, an accurate quantification of circulating miRs from plasma can be applied by standard calibration curve in water.

#### 3.2.2 Sample handling

Although the processing of plasma is largely standardized, further handling is not. In high‐volume stored plasma which was frozen within eight hours of collection (fresh frozen plasma, FFP) and thawed slowly on ice, almost insoluble precipitates can be observed (Figure 2C).^31^ The smaller the storage volume, the less clearly the precipitates can be recognized. However, it cannot be ruled out that precipitates are also present in small storage volumes. Therefore, we tested two methods for processing FFP regarding their influence on the quantification of miRs. On one hand, we tried to homogenize by thoroughly mixing the precipitate in FFP or one the other hand we centrifuged the FFP to separate the precipitates and use the supernatant for the measurements. After separation of precipitates in K3‐EDTA plasma (Figure 2D) and Na‐Citrate plasma (Figure 2E) we observed significant lower levels of circulating miR‐146a‐5p and miR‐382‐5p. The separation process of serum samples did not influence the levels of both miRs (Figure 2F). Contrary, the levels of miR‐155‐5p and miR‐451a were not influenced by separation in considered plasma samples (Figure 2D‐F). To avoid the loss of possibly bound miRs in precipitates of FFP, we have proposed for whole plasma miR analysis portioning of fresh plasma in volumes that can be used directly for RNA extraction to avoid an additional freeze‐thaw cycle.

#### 3.2.3 Sample storage

**Figure 3:**
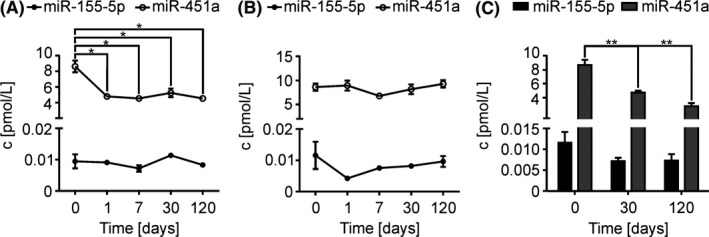
Differential stability of miR‐155 and miR‐451a in plasma, as RNA‐isolate and as cDNA derivative. Evaluation of miR‐155‐5p and miR‐451a stability from 10 µL K3‐EDTA plasma in stored (A) RNA, isolated by Phenol/GTC RNA‐extraction, (B) corresponding cDNA and (C) plasma. MiRs were quantified by RT‐qPCR with SYBR® Green assay and normalized using internal standard cel‐miR‐39‐3p (50 nmol/L). RNA and plasma were stored at −80°C and cDNA at −20°C. MiR‐155 and miR‐451a in RNA samples and as cDNA derivate were analyzed after one day, 7, 30, and 120 d of storage. In plasma samples miRs were quantified from fresh plasma, and after 30 and 120 d of storage. Concentrations of miRs are given as mean + SEM of three independent RNA isolations; *t* test, *P < 0.05, **P < 0.01

The stability of miRs and cDNA is of great importance for reproducible analytical methods. It is generally assumed that isolated miRs and the cDNA are highly stable. However, Bravo et al. could show for the first time that miRs, for example, miR‐451a is highly unstable also as cDNA compared to other miRs.^32^ Therefore, we investigated the storage stability focused on two miRs, miR‐155‐5p and miR‐451a, in plasma, as RNA‐isolate and as cDNA after different storage conditions. After preparation of fresh plasma samples, miR levels were determined during the storage as RNA isolate (Figure 3A) and cDNA derivative (Figure 3B) at different time points (1, 7, 30, and 120 days). No significant variations in miR‐155‐5p concentrations of RNA‐isolates or cDNA were observed over the storage period. Stored as RNA‐isolate, a significant decrease of 50% of miR‐451a concentration was detected within one day of storage (Figure 3A). A decrease in miR‐451a concentrations during storage as cDNA was not observed (Figure 3B). MiR levels were quantified over time in stored plasma (0, 30, and 120 days) (Figure 3C). Even in plasma, we observed no changes in miR‐155‐5p level after the defined storage time. For miR‐451a level we detected a significant decrease after 30 days of storage. Normalization was performed using internal standard cel‐miR‐39‐p. For this purpose, the stability of the internal standard was also investigated, with no significant instabilities of cel‐miR‐39‐3p stored as RNA isolate and cDNA derivative being observed (Figure S3). These first stability tests give a first insight which must be further investigated with extended stability studies of more miRs and with fresh plasma from different volunteers.

### 3.3 The suitability of the internal standard for normalization

**Figure 4:**
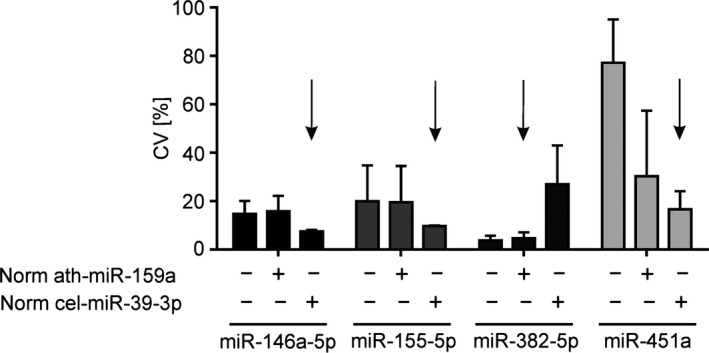
Normalization of technical and methodical variations using different internal standard miRs. Quantification of miRs from 10 µL K3‐ETDA plasma spiked with synthetic miR‐146a‐5p (2.5 nmol/L), miR‐155 (2.5 nmol/L), miR‐382‐5p (2.5 pmol/L) and miR‐451a (250 pmol/L) and normalized using ath‐miR‐159a (50 nmol/L) or cel‐miR‐39‐3p (50 nmol/L) as internal standard miR. The calculated coefficient of variation (CV) from results normalized with the corresponding internal standard is indicated by +, without normalization is indicated by −. CV is given as mean + SEM of three independent RNA isolations on two independent experimental days. The preferred internal standards for normalization are highlighted with arrows

We have tested whether the choice of the internal standard for normalization of technical and methodical variability of miR isolation and RT‐qPCR does not affect the precision (CV, n = 6) of the analyzed miRs. To verify the suitability of the internal standards ath‐miR‐159a and cel‐miR‐39‐3p for normalizing the measured values of miR‐146a‐5p, miR‐155‐5p, miR‐382‐5p, and miR‐451a, the precision (CV) of the measured data was determined. Therefore, RNA was isolated from K3‐EDTA plasma spiked with synthetic miR‐146a‐5p (2.5 nmol/L), miR‐155‐5p (2.5 nmol/L), miR‐382‐5p (2.5 pmol/L), miR‐451a (250 pmol/L) together with ath‐miR‐159a (50 nmol/L) and cel‐miR‐39‐3p (50 nmol/L), respectively and analyzed by RT‐qPCR. The CV of the miR‐146a‐5p, miR‐155‐5p, and miR‐451a concentrations is decreased by normalization with cel‐miR‐39‐3p as internal standard but not by ath‐miR‐159a (Figure 4). Contrary, the CV of miR‐382‐5p quantification was increased by normalization with cel‐miR‐39‐5p. Therefore, the technical and methodical variations of quantified miR‐382‐5p can be normalized with ath‐miR‐159a as internal standard. Our findings indicate that a universal internal standard for normalization of technical and methodical variability of RT‐qPCR assays is not applicable. In the best case, the internal standard is adapted to the analyzed miR.

### 3.4 Standardized miR quantification complied with GLP

We now aimed to define acceptance criteria and recommendations to ensure reliable and reproducible quantification of miRs in human blood samples. Therefore, the parameters of a validation according to GLP were used to standardize the miR quantification. This includes the determination of the lower limit of quantification (LLOQ), the calibration range of analytical method (Figure S4), the accuracy and precision, as well as the determination of matrix effect and of the stability of the analyte. Limit of detection (LOD) was not determined as we validated a quantitative RT‐qPCR and not a qualitative one.^20^ The validation also included PCR efficiency and the slope of calibration curve. Finally, guidelines regarding repeatability, reproducibility and accuracy of the quantification method were established. On three independent measurements of three experimental days, RNA was isolated from 10 µL human K3‐EDTA plasma by GTC‐based RNA extraction method with subsequent quantification of miR‐146a‐5p, miR‐155‐5p, miR‐382‐5p, and mR‐451a using SYBR^®^ Green assay in RT‐qPCR. The method was validated for small amounts of samples, since clinical material is often highly limited. To assess repeatability, reproducibility and accuracy of miR quantification, quality controls (QCs) with different concentrations were used. For the preparation of the QCs, synthetic miR was spiked in plasma samples based on extracellular miR concentration at medium (Med) and low concentration (Low) and at LLOQ. Precision was determined of the whole extraction and analysis process, so samples from one plasma pool were extracted and reverse transcribed in triplicates. The RT‐qPCR analysis was performed in duplicates from each of the processed plasma samples. The resulting CV (n = 3 per day and concentration) determined for intraday precision of the four quantified miRs on each three test days were within 25%, expect for the LLOQ. Here, the CV (n = 3 per day) expended to almost 30% (Table 1). The interday precision of measured concentrations (CV, n = 9) was determined to be within 30%. For the LLOQ the CV was close to 40%. The accuracy of QCs spiked with low concentrations did not fulfill the acceptance criteria. For this a minimum measurable difference between the concentration of spiked synthetic miR and the endogenous analyte concentration is required. The accuracy of QCs with higher concentrations (Med) is approximately ± 25%. The linearity of calibration curves for miR concentrations was provided by R^2^ ≥ 0.98 (Table S1). The RT‐qPCR efficiency calculated from the slope of the standard curve varies between 80% and 109% depending on the analyzed miR. For the quantification of miRs the efficiency varies within ± 10% of the mean of calculated efficiency of three standard curves (Table S1). Furthermore, the accuracy of the back‐calculated concentrations of calibration standards was determined, which varies within ± 30% expect of the standard curves with a correlation coefficient (*R* ^2^) under 1.00 (Table S2). The mean of CV for quantified concentrations of three independent prepared and measured calibration standards are within 15% when *R* ^2^ is approx. 1. When *R* ^2^ is lower one, but still within the generally acceptance criteria of calibration curves for RT‐qPCRs,^20^ the CV is up to 30% (Table S3).

**Table 1:** Intraday‐ and interday‐precision of quantified miRs concentrations from human K3‐EDTA plasma using RT‐qPCR

	Intraday precision CV^a^ [%]			Interday precision (3 days) CV [%]	Intraday accuracy [%]
miR‐146a‐5p					
QC^b^ LLOQ^c^	6.56	8.93	14.39	26.32	rae^d^
QC Low^e^	11.91	3.06	11.00	12.33	rae
QC Med^f^	10.58	5.23	7.84	7.86	6.05
miR‐155‐5p					
QC LLOQ	11.81	6.35	3.43	31.80	rae
QC Low	9.62	11.53	3.64	14.77	rae
QC Med	10.68	1.51	9.11	16.15	2.72
miR‐382‐5p					
QC LLOQ	27.71	13.81	29.60	37.35	rae
QC Low	17.38	15.48	17.80	27.96	rae
QC Med	4.67	9.62	3.40	25.76	12.52
miR‐451a					
QC LLOQ	8.44	13.95	4.68	20.77	rae
QC Low	2.81	10.67	3.23	24.29	23.68
QC Med	9.48	16.06	20.46	19.11	22.82

Note

Precision and accuracy are determined by quantification of quality controls (QC) with specific miR concentrations using SYBR^®^ Green assay by RT‐qPCR. The concentrations of QCs for the quantification of miR‐146a‐5p and miR‐155‐5p are as follows: 25 fmol/L (LLOQ), 250 fmol/L (Low), 25 pmol/L (Med). The following applies to QC of miR‐382‐5p: 2.5 fmol/L (LLOQ), 25 fmol/L (Low), and 2.5 pmol/L (Med). And for QCs of miR‐451a: 250 fmol/L (LLOQ), 2.5 pmol/L (Low), 250 pmol/L (Med). The given CV was calculated for concentrations, which are about 10 times the CV of Cq‐values. Intraday precision of miR quantification is given as CV of three different RNA isolations (n = 3). Interday precision is given as CV, and interday accuracy of miR quantification is given as mean of three independent experimental days (n = 3 per analytical day, ie, n = 9 for each concentration). Variations are normalized using ath‐miR‐159a (50 nmol/L) or cel‐miR‐39‐3p (50 nmol/L) as internal standard.

a Coefficient of variation.

b Quality control.

c Lower limit of quantification.

d Range of acceptance is exceeded.

e Low concentration.

f Median concentration.

Based on our results, we defined acceptance criteria for quantification of miRs in human plasma (Table 2). The PCR efficiency must be 100 ± 10% when quantifying absolute concentrations of RNA by RT‐qPCR using SYBR^®^ Green assay, as it is the case in general acceptance criteria for RT‐qPCR.^20^ For semi‐quantification a PCR efficiency of ±10% of the mean of batches to be compared is acceptable. The CV as intraday precision of quantified concentration of plasma samples processed in replicates must be within 25% (LLOQ 30%) and as interday precision within 35% (LLOQ 40%). The CV of measured Cq‐values has no significance, because Cq is strongly dependent on PCR efficiency. The trueness of QCs or calibration standards depends on R^2^. A correlation coefficient of the calibration curve of ≥ 0.98 is acceptable but for an accurate quantification of absolute concentration the *R* ^2^ must be approximately 1.00. The base lies in the documentation of the described PCR parameters appropriate for GLP standards. To ensure reliable data, all these parameters need to be documented and presented in publications of scientific work. These acceptance criteria were defined according to GLP and the extent to which they are used in general laboratory routine for biomarker studies needs to be evaluated. Even though we initially only validated miR analysis on unfractionated samples, our analysis can be adapted to miRs in extracellular vesicles (Figure S5).

**Table 2:** Acceptance criteria and recommendations for miR quantification using SYBR^®^ Green assay in RT‐qPCR

Parameter	Acceptance criteria	Recommendations/ comments
PCR Efficiency	90%‐110%	Absolute quantification: 100 ± 10% Semi‐quantification (eg, comparative quantification of extracellular miR level): ±10% of the mean of batches to be compared; mean does not necessarily have to be 100%
Slope of standard curve	−3.1 to −3.6	Slope up to −4.1 is acceptable, when it is constant within batches to be compared
Correlation coefficient (R^2^)	≥ 0.98	Correlation coefficient lower than 1 result in inaccuracy quantification of at least one calibration standard
Linearity	see *R* ^2^	Within calibration range of extracellular miR concentrations; A^a^ [%] of calculation standards ± 30%, but can be higher when *R* ^2^ < 1.0
Specificity	Tm^b^ ± 1°C	Melting curve analysis of all batches
Sensitivity	A_LLOQ_ c ≤ 30%	Accuracy of standard curve can be higher when R^2^ < 1.0
Repeatability	CV^d^ ≤ 25% CV_LLOQ_ ≤ 30%	CV of quantified concentrations, not Cq‐values
Reproducibility	CV ≤ 35% CV_LLOQ_ ≤ 40%	CV of quantified concentrations, not Cq‐values
Trueness	A_QC_ ± 30%	Accuracy of low QCs^e^ is not within acceptance criteria

a Accuracy.

b Melting temperature.

c Lower limit of quantification.

d Coefficient of variation.

e Quality control.

## 4 DISCUSSION

Circulating miRs are certainly one of the most potential biomarkers that may aid risk assessment, diagnosis,^33^, ^34^ prognosis,^35^, ^36^ and monitoring of individual treatment response^37^ which is supported by the high number of new publications each year. Despite growing enthusiasm for the use of such a liquid biopsy marker, there are still a number of challenges in this area. As reviewed by Witwer KW the reproducibility of biomarker studies is almost not existent which he attributed to the non‐standard terms of the studies.^10^ Therefore, we decided to validate miR analysis by RT‐qPCR using the GLP standards. We analyzed miR‐146a‐5p, miR‐155‐5p, miR‐382‐5p, and miR‐451a, which have been described as potential blood‐based biomarkers for various diseases.^21^, ^22^, ^23^, ^24^ For the validation of miR analysis we considered methodological differences in RNA extraction and isolation. Here, we compared a simple and cost‐efficient phenol/GTC method according to Chomczynski & Sacci (1987)^13^ and a column‐based RNA extraction. In addition to the higher recovery of miRs isolated by phenol/GTC method with subsequent precipitation, a dependence of the recovery rate on the miRs was observed. As the RNA extraction of both methods is based on phenol, the purification has to be considered as a reason for differences in recovery. The inconsistency of the recovery rate of column‐based RNA purification was associated with sequence differences of miRs.^15^ Variations in the short miR sequence lead to explicit different secondary structures, leading to different precipitation levels in phenol/GTC RNA extraction.^38^ In order to avoid the influence of different extraction efficiencies on absolute miR quantification, we treated calibration standards like plasma samples with phenol/GTC method. While the phenol/GTC method with subsequent ethanol precipitation for analyzing circulating miRs is more cost‐efficient and higher in yield than tested column‐based RNA isolation methods, this miR isolation method is not applicable for full automatization process. For this purpose, further development of the method is necessary.

MiR expression levels differ considerably between serum and plasma samples.^39^ Timms et al have already described the release of cellular components into serum by cells in the clot during coagulation for more than 60 minutes.^40^ Considering this argument in combination with better standardization of plasma processing, we recommend the use of plasma for biomarker studies. When analyzing the impact of pre‐analytical variables of miR analysis, we found that the level of circulating miRs were influenced by differential sample handling, such as the portioning of plasma samples from larger sample volumes. When stored plasma samples are slowly thawed at 4°C, cryoprecipitates are formed which contain blood clotting proteins^41^ and a high content of microparticles.^42^, ^43^ The level of miR‐146a‐5p and miR‐382‐5p is dependent on the portioning of plasma with or without portions of cryoprecipitate. Our results suggest that the level of circulating miRs which either interact with proteins or are packed in extracellular vesicles is influenced by cryoprecipitation, leading to variations in the miR quantification of unfractionated plasma. Therefore, every step of the circulating miR analysis should be standardized to avoid unspecific deviations. In this case, we recommend for whole plasma miR analysis portioning fresh plasma into the final analysis volume.

There is no universally valid endogenous reference miR known to be sufficient to normalize the miR level in the body fluid.^44^ Therefore, we used internal standards of non‐human origin like ath‐miR‐159a and cel‐miR‐39‐3p which have been used as general internal standards for normalization in miR‐analysis.^11^, ^45^ We found disparities in quantification results of different miRs, depending on the choice of internal standard. We could show that normalizing with an inappropriate internal standard lead to inconsistent miR quantification. The underlying cause of fluctuations in normalization results will be further investigated in order to find a strategy for predicting appropriate internal standards for specific analysis of miRs. With regard to the standardization of miR analysis, the suitability of such internal standards should be verified and documented in validation of specific miR biomarkers.

We first introduce acceptance criteria for monitoring the performance of miR analysis form plasma and to ensure reliability of RT‐qPCR data. The acceptance criteria refer to the determination of the concentrations of circulating miRs. The Cq‐values may vary between the different RT‐qPCRs, depending strongly on the PCR efficiency. For example, Cq‐values are only reproducible to a limited extent without information on PCR efficiency. Here, the standards of analytical work under GLP were adopted. All analyses are verified by calibration standards and quality control samples to ensure comparable efficiencies of the individual RNA isolations and RT‐qPCRs and to check the variability of all analyzed data. With this validated method it is possible to accurately assess the stability of miRs in the stored plasma and after RNA isolation.

Based on our validation results, we consider a basic verification of miR quantification results as essential, especially with regard to repeatability and reproducibility of miR isolation and analysis (Table 2). Related to future experimental and clinical investigations of miR biomarkers we highlighted here recommendations and general implementations related to GLP principles, that need to be considered when analyzing circulating miRs.

## CONFLICT OF INTEREST

The authors DJK, LS, and MF are employees of Prolytic GmbH. As a bioanalytics company, Prolytic GmbH will commercially offer the method presented as a service. MJS is currently scientific advisor of Prolytic GmbH. The other author declares no conflict of interest.

## AUTHOR CONTRIBUTIONS

MF performed experiments, analyzed the data, and wrote the manuscript. ABH and LS performed experiments and analyzed data. DJK contributed to writing the manuscript and designed the project. MJS conceived the study, designed and supervised the overall project, and wrote the manuscript. All authors conducted the quality assurance of the paper and reviewed the manuscript.
