# Supplementary material for: Validation of extracellular miRNA quantification in blood samples using RT‐qPCR
Source: FASEB Bioadv. 2019 Jul 1;1(8):481–92. doi: 10.1096/fba.2019-00018 (PMC6996320; doi:10.1096/fba.2019-00018)
Supplement: Supplementary file 1 [file FBA2-1-481-s001.pdf]

Supplementary Information

Validation of extracellular miRNA quantification in blood samples using RT-qPCR

Maria Fauth [1,2], Anett B. Hegewald [1], Lisa Schmitz [2], Dorothee J. Krone [2], Meike J. Saul [1,3*]

[1] Department of Biology, Technische Universität Darmstadt, Schnittspahnstraße 10, 64287 Darmstadt, Germany

[2] Prolytic GmbH, Weismüllerstraße 45, 60314 Frankfurt/M, Germany

[3] Institute of Pharmaceutical Chemistry, Goethe Universität Frankfurt, Max-von-Laue-Straße 9, 60438 Frankfurt/M., Germany

* Corresponding author: Meike J. Saul

E-mail: saul@bio.tu-darmstadt.de

Extracellular vesicle (EV) isolation by ultracentrifugation

500 µl plasma sample was diluted 1:2 with PBS (1x DBPS; Gibco, Carlsbad, USA) and centrifuged at 2,000 g at room temperature for 20 min to remove cell debris. The supernatant was transferred to ultracentrifugation tubes (Beckman Coulter Life Science, Lakeview, USA) followed by ultracentrifugation at 21,000 g and 4°C for 60 minutes to remove large membrane vesicles. The supernatant was transferred in a new tube and centrifuged at 100,000 g and 4°C for 60 min. Finally, the supernatant was discarded, and the resulting EV pellet was lysed with 420 µl of lysis buffer for RNA extraction. RNA isolation was performed according to Phenol/GTC RNA extraction method.

Electron microscopy of EVs

For transmission electron microscopy (TEM) EVs from human plasma were purified as described above and resuspended in PBS. A drop of purified EVs was placed on parafilm and a formvar carbon coated nickel grid (Plano, Wetzlar, Germany) was placed on top of the drop for 30-60 min. The grid was washed three times by sequentially positioning the grid on top of droplets of PBS and the use of absorbing paper in between. The samples were fixed with 2% paraformaldehyde (Carl Roth, Karlsruhe, Germany) for 10 min and washed again three times with PBS. Then the EVs were incubated with 2.5% glutaraldehyde for another 10 min and subsequently washed three times with deionized water. To contrast the sample, it was incubated with 2% uranyl acetate for 15 min. The excess liquid was removed by using an absorbing paper and the grid was air dried for 5 min. The EVs were examined by Zeiss EM109 electron microscope.

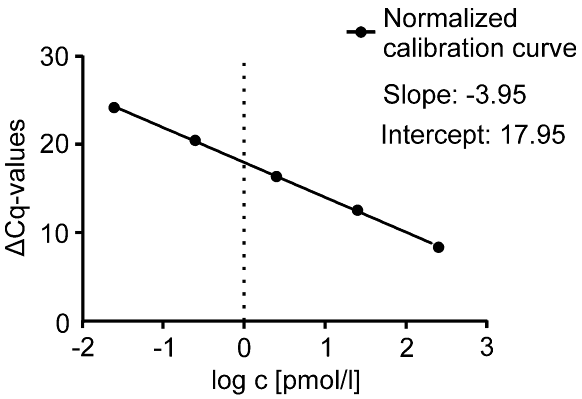

S1 Fig. Normalized calibration curve of synthetic miR-146a-5p diluted in water and quantified by RT-qPCR.

Illustration of a normalized calibration curve, used for the calculation of miR-concentration due to the slope and intercept of calibration curve. Normalization of Cq-values is done by calculation of ΔCq as $\Delta\mathrm{Cq} = \mathrm{Cq}\ (\mathrm{analyte}) - Cq\ (internal\ standard).$ Calibration standards were prepared like plasma samples with GTC-based RNA extraction method and quantified by RT-qPCR.

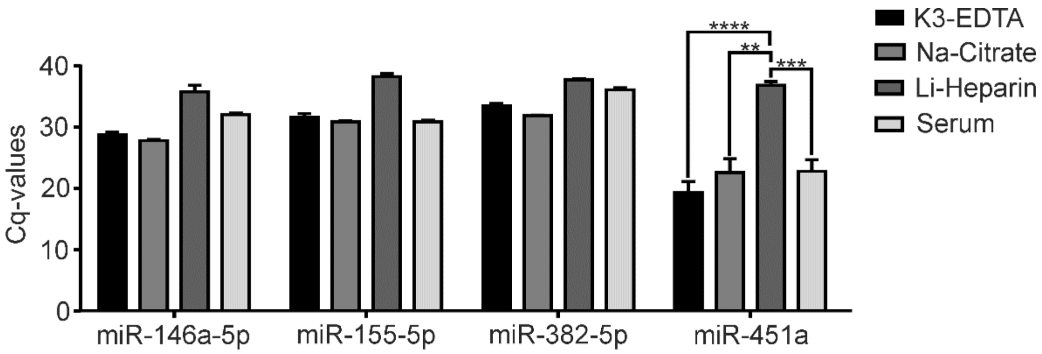

S2 Fig. Quantification of miRs isolated from different matrices by RT-qPCR.

Comparison of Cq-values from quantification of circulating miR-146a-5p, miR-155-5p, miR-382-5p and miR-451a isolated from 10 µl human plasma or serum. The influence of different matrices (serum and plasma with additional consideration of anticoagulants potassium-3-ethylenediaminetetraacetic acid (K3-EDTA), sodium citrate (Na-Citrate) and lithium heparin (Li-Heparin)) on quantification of miRs by RT-qPCR was assessed. There are no significant difference in Cq-values of miR quantification from serum and plasma with anticoagulants K3-EDTA and Na-Citrate. MiRs isolated from Li-Heparin plasma are indeterminable or the Cq-vales are higher than 37 Cq. Cq-values are given as mean + SEM of six independent RNA isolations; t-test, **$p < 0.01$, ***$p < 0.001$, ****$p < 0.0001$.

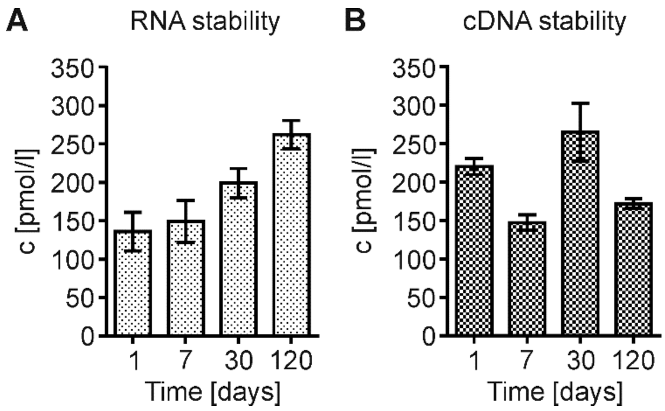

S3 Fig. Stability of cel-miR-39-3p as RNA-isolate and as cDNA derivative.

Stability investigations of cel-miR-39-3p stored in (A) RNA samples and (B) as corresponding cDNA derivate. RNA was stored at -80°C and cDNA at -20°C. Cel-miR-39-3p was quantified by RT-qPCR with SYBR® Green assay after one day, 7 days, 30 days and 120 days of storage following one freeze/thaw cycle. Concentrations are given as mean + SEM of three independent RNA isolations.

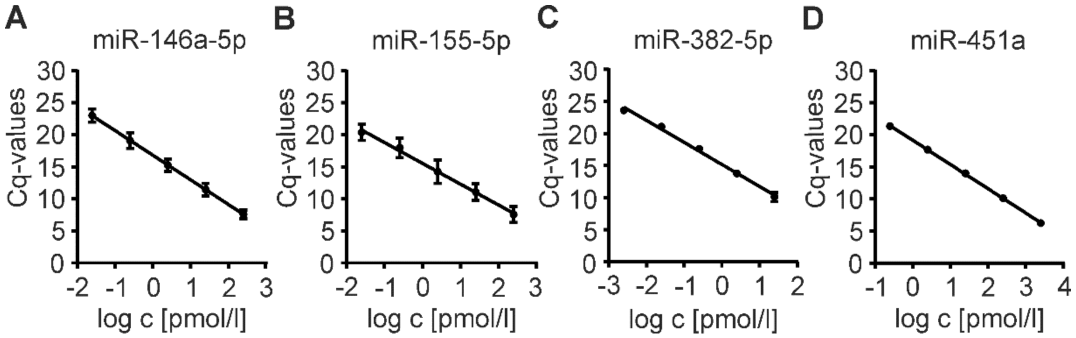

S4 Fig. Standard calibration curves of synthetic miRs diluted in water and quantified by RT-qPCR.

The linearity of the standard calibration curve to determine concentration of (A) miR-146a-5p, (B) miR-155-5p, (C) miR-382-5p and (D) miR-451a was evaluated by serial dilution of the synthetic miRs in RNase-free water over 5 orders of magnitude. The standard calibration curves include the range of the circulating target miR level in human plasma. Calibration standards were prepared like plasma samples with GTC-based RNA extraction method and quantified by RT-qPCR. Linearity was assessed by preparing and measuring three standard calibration curves on three independent experimental days. The calibration standards are given as mean + SEM.

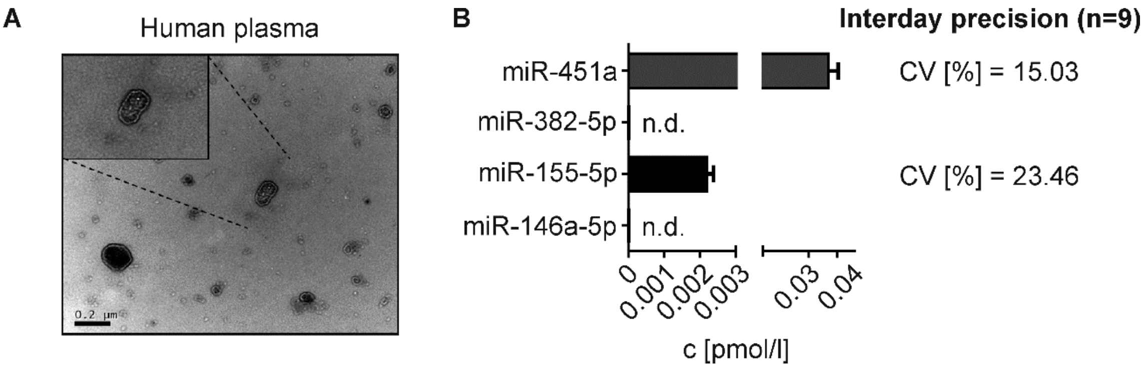

S5 Fig. Analysis of miRs by validated miR quantification method isolated from extracellular vesicles, termed as exosomes.

(A) Transmission electron microscopy (TEM) of extracellular vesicles from human plasma isolated by different ultra-centrifugation steps. Picture is shown from one representative experiment. (B) Exosomal miR-146a-5p, miR-155-5p, miR-382-5p and miR-451a were isolated after ultracentrifugation from 500 µl human K3-EDTA plasma by phenol/GTC RNA extraction method with subsequent ethanol precipitation and quantified by RT-qPCR using SYBR® Green assay. MiR-146a-5p and miR-382-5p could not detected (n.d.), but interday precision of the quantification of exosomal miR-155-5p and miR-451a was within acceptance criteria. Concentrations of miRs are given as mean + SEM of nine independent RNA isolations Aon three independent experimental days.

S1 Table. Parameters of three standard calibration curves (assay 1-3) of synthetic miR-146a-5p, miR-155-5p, miR-382-5p and miR-451a quantified by RT-qPCR.

	Assay	Slope	Intercept	R^2	Efficiency [%]
miR-146a-5p	1	-3.78	16.42	1.00	84
	2	-3.95	17.95	1.00	79
	3	-3.80	16.05	1.00	83
miR-155-5p	1	-3.12	12.80	0.99	109
	2	-3.50	16.17	0.99	93
	3	-3.13	17.63	0.99	109
miR-382-5p	1	-3.27	16.29	1.00	102
	2	-3.63	14.46	1.00	89
	3	-3.39	14.83	0.98	97
miR-451a	1	-3.90	19.43	1.00	80
	2	-3.52	18.23	1.00	92
	3	-3.90	19.79	1.00	80

The efficiency was calculated from slope of the standard calibration curves. The efficiencies of the different standard calibration curves varies from 80 to 109%, but the variation within the standard calibration curves of one miR did not exceeded 20%. The correlation coefficient (R2) of all standard curves is at least 0.98.

S2 Table. Back calculated concentrations (c_norm) and calculated intraday accuracy (A) of three independently prepared and measured calibrations standards of miR-146a-5p, miR-155-5p, miR-382-5p and miR-451a.

	Calibration standard c[§] [pmol/l]	C_norm 1 [pmol/l]	A[§§] [%]	C_norm 2 [pmol/l]	A [%]	C_norm 3 [pmol/l]	A [%]
miR-146a-5p	250	243.32	-2.67	268.27	7.31	235.30	-5.88
	25	25.45	1.82	23.25	-7.00	26.08	4.30
	2.5	2.58	3.30	2.50	0.15	2.46	-1.47
	0.25	0.25	-1.06	0.23	-6.53	0.29	15.69
	0.025	0.025	-1.27	0.027	7.04	0.022	-10.63
miR-155-5p	250	223.60	-10.62	319.18	27.67	312.14	24.85
	25	20.18	-19.30	25.36	1.44	25.72	2.89
	2.5	4.77	rae[§§§]	1.84	-26.50	1.85	-26.10
	0.25	0.20	-18.04	0.17	-33.27	0.17	-30.82
	0.025	0.022	-11.30	0.04	rae	0.04	rae
miR-382-5p	25	27.33	9.33	26.95	7.81	36.25	rae
	2.5	2.82	12.65	2.49	-0.60	2.41	-3.54
	0.25	0.19	-25.62	0.23	-8.60	0.17	-33.22
	0.025	0.02	-11.50	0.023	-9.80	0.014	rae
	2.5×10^{-3}	0.003	23.34	0.003	13.19	0.005	rae
miR-451a	2500	2702.63	8.11	2368.04	-5.28	2778.58	11.14
	250	238.45	-4.61	265.82	6.33	240.64	-3.74
	25	21.82	-12.74	25.22	0.88	23.43	-6.29
	2.5	2.77	10.79	2.54	1.54	2.09	-16.33
	0.25	0.25	0.31	0.24	-3.07	0.30	19.21

[§]: Concentration; [§§]: Accuracy (intraday); [§§§]: Range of acceptance exceeded

Concentrations of miRs are normalized using ath-miR-159a (50 nmol/l) or cel-miR-39-3p (50 nmol/l) as internal standard.

S3 Table. Residuals of normalized Cq-values (Cq_norm) and quantified concentrations (c_norm) of calibration standards of miR-146a-5p, miR-155-5p, miR-382-5p and miR-451a.

	Calibration standard c[§] [pmol/l]	Mean Ct_norm	CV[§§] [%]	Mean c_norm [pmol/l]	CV [%]	A[§§§] [%]
miR-146a-5p	250	7.59	9.06	248.96	6.91	17.62
	25	11.44	8.65	24.93	1.76	-1.70
	2.5	15.27	6.37	2.52	3.35	-1.12
	0.25	19.09	6.37	0.26	11.53	0.04
	0.025	23.00	4.39	0.025	6.73	0.00
miR-155-5p	250	7.57	28.80	284.93	18.72	13.97
	25	11.07	20.33	23.75	13.06	-5.00
	2.5	14.24	22.32	1.84	0.38	-26.30
	0.25	17.95	14.85	0.18	11.27	-27.38
	0.025	20.37	10.57	0.03	28.82	32.79
miR-382-5p	25	10.13	12.53	30.72	17.44	20.72
	2.5	13.79	6.72	2.57	8.39	-22.48
	0.25	17.64	5.46	0.19	16.26	-22.48
	0.025	21.08	3.05	0.020	24.21	-21.60
	2.5 x 10^{-3}	23.64	3.80	3.6 x10^{-3}	29.17	42.00
miR-451a	2500	6.24	2.75	2616.42	8.35	4.66
	250	10.11	3.80	248.31	6.12	-0.68
	25	13.98	4.27	23.49	7.25	-6.05
	2.5	17.68	4.87	2.47	13.97	-1.33
	0.25	21.34	3.83	0.26	11.38	5.48

[§]: Concentration; [§§]: Coefficient of variation; [§§§]: Accuracy (interday, n= 3)

Cq-values and concentrations are given as mean of three independent experimental days. Normalisation was performed using internal standards of the analytes.

S4 Table. Melting temperatures (Tm) of intraday and interday precision analysis of miR-146a-5p, miR-155-5p, miR-382-5p and miR-451a.

	Tm Intraday [C°]			Tm Interday [C°]	Standard deviation
miR-146a-5p	75.99	76.02	75.96	75.99	0.03
miR-155.5p	76.30	76.18	76.23	76.24	0.06
miR-382-5p	76.92	76.84	76.83	76.86	0.05
miR-451a	76.01	76.06	76.19	76.09	0.09
cel-miR-39-3p	76.81	76.78	76.85	76.81	0.04
ath-miR-159a	76.30	76.20	76.29	76.26	0.05
